# In Vitro Generation of Oocyte Like Cells and Their In Vivo Efficacy: How Far We have been Succeeded

**DOI:** 10.3390/cells9030557

**Published:** 2020-02-27

**Authors:** Dinesh Bharti, Si-Jung Jang, Sang-Yun Lee, Sung-Lim Lee, Gyu-Jin Rho

**Affiliations:** 1 Department of Theriogenology and Biotechnology, College of Veterinary Medicine, Gyeongsang National University, Jinju 52828, Korea; bhartidinesh54@gnu.ac.kr (D.B.); sjjang@gnu.ac.kr (S.-J.J.); sy_lee@gnu.ac.kr (S.-Y.L.); sllee@gnu.ac.kr (S.-L.L.); 2 Research Institute of Life Sciences, Gyeongsang National University, Jinju 52828, Korea

**Keywords:** mesenchymal stem cells, primordial germ cells, oocyte-like cells, oogenesis, differentiation, transfection, folliculogenesis

## Abstract

In the last few decades, stem cell therapy has grown as a boon for many pathological complications including female reproductive disorders. In this review, a brief description of available strategies that are related to stem cell-based in vitro oocyte-like cell (OLC) development are given. We have tried to cover all the aspects and latest updates of the in vitro OLC developmental methodologies, marker profiling, available disease models, and in vivo efficacies, with a special focus on mesenchymal stem cells (MSCs), induced pluripotent stem cells (iPSCs), and embryonic stem cells (ESCs) usage. The differentiation abilities of both the ovarian and non-ovarian stem cell sources under various induction conditions have shown different effects on morphological alterations, proliferation- and size-associated developments, hormonal secretions under gonadotropic stimulations, and their neo-oogenesis or folliculogenesis abilities after in vivo transplantations. The attainment of characters like oocyte-like morphology, size expansion, and meiosis initiation have been found to be major obstacles during in vitro oogenesis. A number of reports have either lacked in vivo studies or have shown their functional incapability to produce viable and healthy offspring. Though researchers have gained many valuable insights regarding in vitro gametogenesis, still there are many things to do to make stem cell-derived OLCs fully functional.

## 1. Introduction

In the last few decades, the in vitro development of oocyte-like cells (OLCs) with the ability to cure female reproductive disorders by neo-oogenesis and folliculogenesis has been the main focus of reproductive and stem cell scientists. Researchers have succeeded in deriving primordial germ-like cells (PGCLCs), follicle-like cells (FLCs), and OLCs, as well as in treating animal disease models by using embryonic stem cells (ESCs), induced pluripotent stem cells (iPSCs), and neonatal and adult ovarian stem cell sources from different species including humans [1,2,3,4,5,6,7,8,9,10,11,12,13,14,15,16], mice [17,18,19,20,21,22,23,24], porcine [25,26,27,28,29], bovines [30,31], and goats [32]. Ovaries are the primary source and reservoir of oocytes. Attempts have been made to isolate cells from different parts of ovaries by using different methodologies. Researchers have succeeded in isolating cells from the ovarian surface epithelium (OSE) and the ovarian cortex, and they have also utilized whole ovaries by using mechanical and enzymatic degradation methods [1,22,23,29,31,33,34,35]. By culturing these cells in specific media or inducing the ectopic expression of some transcriptional factors, OLCs that not only attained oocyte-like morphology but exhibited germ cell-specific marker expression both at messenger ribonucleic acid (mRNA) and protein levels have been generated [29]. This approach of inducing ectopic expression by transfection or viral inductions had also proved efficient in developing OLCs from non-ovarian stem cell sources [7,16,28]. During oogenesis (in vivo), a fluid-like substance enriched with a variety of molecules such as proteins, polysaccharides, steroid hormones, growth factors, reactive oxygen species, and many such valuable components is secreted by growing antral follicles that surround the developing oocyte and also helps in follicular maturation by providing a conducive microenvironment [36]. To mimic this supportive niche, many researchers have tried to enrich their differentiation media by using different concentrations (ranging from 5%–25%) of human-, bovine-, and porcine-derived follicular fluid (FF) alone or in combination with other differentiation inducers such as bone morphogenic protein (BMP) family members, retinoic acid (RA), and Activin-A, and succeeded in getting PGCLCs, FLCs or OLCs [12,29,31,37]. Stem cells have the ability to differentiate into many lineages when cultured under lineage-specific culturing conditions, for which selection of the stem cell source is equally important as the selection of the differentiation protocol. Moreover, different mesenchymal stem cell (MSC) sources may have different developmental potencies for targeted lineages. Therefore, before starting any lineage-specific differentiation, the selection of the most suitable cell source is highly recommended. Keeping this necessity in mind, many groups have compared the differentiation ability of MSCs that were derived from various sources when using the same induction conditions [38,39,40]. From the growing body of literature, one such source with intrinsic germ cell differentiation potential has been found to be fetal or neonatal skin-derived stem cells. Researchers have claimed that skin-derived stem cells (SDSCs), which have a high potency for OLC differentiation, not only resemble oocyte-like morphology and exhibit a higher expression level of oocyte-specific markers but also possess hormonal secretion under gonadotropic stimulations [13,25,26,30]. Oocyte-specific developmental and functional competency also depend on the presence of growth-promoting and healthy cells (e.g., granulosa, theca and cumulus cells) surrounding the growing oocyte. Their presence is vital for the growth of the oocytes. To fulfil this requirement, researchers have adopted co-culturing strategies to derive OLCs in vitro from various stem cell sources. By using multistep differentiation approach, researchers have used cells that were cultured with specific differentiation mediums and plated onto inactivated mouse embryonic fibroblast (MEF) layers. Furthermore, different source-derived stem cells were either transformed into embryoid bodies (EBs) and/or induced with differentiation promoters along with granulosa cell (GC) co-culturing [12,15,20,22,41]. This approach has resulted in the derivation of OLCs with comparable properties like natural oocytes. Their pluripotent nature and ability to differentiate into any cell type makes ESCs the master of all the stem cells. However, due to ethical concern and safety issues, their use has been neglected. However, researchers have been deeply concerned with finding alternative stem cell sources with comparable features. Induced pluripotent stem cells (iPSCs) have provided a solution to this problem, but these cells also have similar demerits to ESCs. Despite these concerns, OLCs have been developed from ESCs and iPSCs [16,20,24,32,42], and continuous efforts are in progress to eradicate safety issues that are related to the use of ESCs and iPSCs. One such cell source with early developmental cell-like properties is that of very small embryonic-like stem cells (VSELs). VSELs have been utilized to derive OLCs in vitro, and micro-RNAs (miRNA) from VSELs have been shown to have the peculiar ability to treat female reproductive disorders like premature ovarian failure (POF) and premature ovarian insufficiency (POI) [43,44]. Despite these wonderful properties, VSELs have also been found to survive chemotherapeutic applications, which makes them a more valuable cell source [45]. However, due to the sophisticated isolation methods that are needed, confirmation criteria and very low cell yield, VSELs usage at large scale is limited and will need more time for proper exploration. Despite all these developments, the direct use of stem cells (undifferentiated state) have also revealed impressive results in treating disorders like POF and POI due to their low immunogenicity, paracrine signals and homing abilities [46,47,48,49,50,51,52,53,54,55,56]. Additionally, researchers throughout the world are putting in their best efforts to standardize differentiation protocols to get safer and more desirable results. However, before looking forward for their use in human beings, many efforts are still needed to succeed in developing fully functional and viable offspring from these in vitro differentiated OLCs. In this review, we tried to mainly focus on discussing the available strategies to develop female germ cells in vitro.

## 2. Ovarian Stem Cells (OSCs) Isolation and Their Differentiation Potential

A number of researchers have documented the presence of valuable stem cells in the ovaries that have been derived from different species including humans, porcine, mice, and bovines. [27,29,31,57]. Different approaches that have been adopted to isolate these cells include the use of ovarian cortex fragments [29,57], the scraping of the ovarian surface epithelium (OSE) [1,2], fluorescence-activated cell sorting (FACS) [5,6,23,58], magnetic-activated cell sorting (MACS) [5,33,34,59,60], and isolation on the basis of cell morphology [22,61].

Researchers have also focused on evaluating the oocyte-specific features from different reproductive aged women-derived OSCs. A recent study on DEAD(Asp-Glu-Ala-Asp)-box polypeptide-4 (DDX4)-positive OSCs that were derived from non-menopausal and menopausal women revealed that OSCs, that were derived from different sources had the ability to differentiate into large haploid OLCs and could express oocyte-specific markers, and could also enter meiosis when cultured under appropriate culturing conditions [57]. Interestingly, different-sized (small and large) cells were isolated from both non-menopausal and menopausal women, which showed high positivity for immature oogonial ontogenetic markers, i.e., stage-specific embryonic antigen 4 (SSEA-4) and interferon-inducible gene commonly known as FRAGILIS. Upon in vitro culturing, large-sized cells exhibited positive expression for mature oocyte-specific markers such as Gdf9 and Scp3, whereas only small-sized cells could express Dppa3 (primordial germline marker) but lacked mature markers, indicating their immature status. Despite having a difference in the number of SSEA-4^+^ and FRAGILIS^+^ subsets of DDX4-positive populations from both OSCs, few differences were observed in their ability to express oocyte-specific markers. Therefore, for targeting OLC differentiation, the size of the isolated cells should also be considered. Moreover, another report from porcine-derived OSCs demonstrated that OLC differentiation potential in vitro and ovarian follicle formation in vivo can be enhanced by inducing the ectopic expression of Oct4 in OSCs [29]. Oct4 is an important pluripotency marker which is also categorized as a germ cell marker. OSE scrapings have also been used to isolate OSCs [1,2,5]. The OSE does not have naturally present oocytes and follicles, but cells can be isolated by scraping the surface epithelium, and these cells can be separated by density gradient centrifugation and proliferated by culturing under specific conditions. OSE cells that are isolated from menopausal as well as premature ovarian failure (POF) patients were found to have a 2–4 µm diameter that gradually increased with the culture expansion and attained a size of about 90 µm at the 20th day of culture and finally transformed into OLCs. OSEs (immediate after scraping) highly expressed Oct4A, Oct4B, Sox2, Nanog and c-Kit transcription markers whose expression was retained even after 20 days (except Nanog and Sox2). After culturing for 20 days, these cells were also found to be positive for germ oocyte-specific transcription factors including Vasa and Zp2, whereas there was no expression for Scp3, which indicates their immature nature. Additionally, isolated cells showed an increased population of SSEA-4-positive cells ranging from 10% to 32% from fresh scrapings and 20-day-cultured cells, respectively [2]. From the above outcomes, it was concluded that OSE scraping-derived cells have the potential to undergo OLC differentiation, irrespective of the reproductive status of the donor, whereas high expression of pluripotency markers such as SSEA-4 and Oct4 explains their embryonic-like cell nature. SSEA-4, Oct4 (embryonic stem cell-like features) and c-Kit (primordial germ cell ancestry) expression can be correlated with the developmental potential of OSE cells towards OLC generation. These markers also play a very important role during early folliculogenesis (under natural conditions). Therefore during stem cell selection for OLC differentiation, they can be considered to be valuable markers. By using both FACS and MACS techniques, Virant-Klun and colleagues reported the successful isolation of small SSEA-4-positive stem cells by the brushing of an OSE of reproductive age, post-menopausal and premature ovarian failure (POF) adult ovaries that were devoid of any carcinogenic history. SSEA-4-positive OSE cells, when cultured with a medium that was supplemented with 20% FF, resulted in an expanded size with a large diameter. In accordance with their previous findings, it was concluded that small putative stem cells can be isolated from all reproductive age, POF females, as well as post-menopausal women. OLCs that were derived from the OSE cells of adult ovaries positively expressed synaptonemal complex protein 3 (SCP3) along with other pluripotency and primordial germ cell-specific markers, but they did not show expression for VASA (a member of DEAD box family, RNA binding protein). The expression of SCP3 may be attributed to the FF that was used in this study, whereas the absence of VASA might have been due to their early and immature oogonial-like stem cell stage [5].

The isolation of OSCs has remained the controversial issue. Most of the FACS-based ovarian stem cell isolation protocols have been primarily associated with the immuno-detection of DDX4-positive cells. As per the reports by Ji Wu and colleagues, the possibility of FACS-based DDX4-positive cells can be attributed to the presence of the externally putative extracellular epitope of Ddx4 on OSC surfaces [33]. Mouse and human OSCs that have been isolated by this technique have been found to be homogenous, pure, and morphologically identical, as well as to possess similar genetic signature-like primitive germ cells. Recently, the Telfer group advocated for the utilization of DDX4-antibody-based FACS to isolate putative OSCs [62]. To target N- and C-terminal epitope tags, a novel DDX4 expression construct was designed to investigate the cellular localization of DDX4. Overall results supported OSCs isolation by FACS by using the antibody-based DDX4 detection method. However, some of the recent reports have shown contradicting results while demonstrating that DDX4 antibody-based FACS-isolated OSCs neither expressed any DDX4-specific mRNA expression, as evaluated by RT-PCR, nor exhibited any *DDX4* expression (mRNA) by single-cell mRNA sequencing [63]. Similar findings have been observed by many other researchers [64,65,66]. From these reports, it is clear that the isolation of OSCs by DDX4 antibody-based FACS isolation is controversial, and other more appropriate and trustworthy methods are therefore required. Researchers have identified and confirmed the presence of female germline stem cells (FGSCs) by utilizing a dual immunofluorescence analysis technique in which five-day-old female mice ovaries were injected with 5′-bromodeoxyuridine (BrdU), and the presence of BrdU–mouse VASA homolog (MVH) double positive cells were further evaluated from OSE cells by using MACS [33]. Isolated cells were cultured for more than 14 days, and it was found that almost all of the cells showed positivity for BrdU and MVH. Interestingly, cells maintained the characteristic morphology of freshly isolated FGSCs even after 25 passages and culturing for more than six months. These features were also successfully maintained even after cryopreservation and thawing. When green fluorescent protein (GFP)-transfected FGSCs were transplanted into the infertile mice ovaries, viable offspring that showed the GFP transgene were produced. An FGSC line from the neonatal mice (nFGSCs) was maintained for about 15 months without any complications. Both the adult- and neonatal-derived FGSCs showed a positive expression for Dazl, Mvh, Blimp1, Oct4, Fragilis, Scp and Zp3 at mRNA and protein levels. FGSC isolation efficiency was further improved by utilizing Fragilis-based magnetic sorting [34]. In spite of the above-mentioned protocols for isolating OSCs, one easier and economical method was reported by Parvari and colleagues [22,61]. This method demonstrated the isolation of OSCs from postnatal mouse ovaries on the basis of cell morphology. In this paper, five-to-seven-day-old neonatal ovaries were dissected, processed, and transferred to a tube to avoid fibroblast contamination. Buoyant cells from the tube were transferred onto gelatin-coated culture dishes by using Dulbecco’s modified eagle’s medium (DMEM) that was supplemented with β-mercaptoethanol, non-essential amino acids, L-glutamine, a penicillin and streptomycin mixture, leukemia inhibitory factor, and fetal bovine serum (FBS), and then these dishes were maintained for 7–10 days. Finally, a growth of small colonies with typical morphology was observed. These colonies were further transferred onto the inactivated MEF layer. Cells from these colonies showed alkaline phosphatase (AP) activity and also exhibited positive expression for octamer-binding transcription factor 4 (OCT4), SSEA-1, MVH and deleted in azoospermia like (DAZL) proteins through immunocytochemical staining. Cells were also found to be positive for Oct4, Nanog, Fragilis, C-kit, Mvh, Dazl, Scp3 and Gdf9 when evaluated by RT-PCR. Earlier, the same approach was utilized to isolate testicular cells [67]. The functional details of pluripotency, PGC, germ cell, meiosis, oocyte, granulosa and theca cell markers are given in Table 1.

## 3. Ectopic Expression/Overexpression of Germ Cell-Specific Markers

With advancements in cell reprogramming and engineering techniques, different cell lineages have been generated by inducing the ectopic expression of lineage-specific transcription factors [68,69,70]. Many researchers have also used this distinctive idea to generate OLCs from various ovarian and non-ovarian stem cell sources that were derived from different species [7,11,16,29,32,42,71]. Oct4, which is one of the main transcription factors that is associated with the pluripotency of stem cells, has also been associated with germline cells and is known as germ cell marker [72]. When utilizing porcine ovarian stem/stromal cells (OvSCs), the overexpression of Oct4 has resulted in the enhancement of OSCs towards OLC differentiation in vitro, as well as ovarian follicular formation in vivo [29]. Porcine OvSCs, when exogenously transfected with Oct4, have been found to not only enhance AP activity and proliferation potential but also to result in higher oogenesis potential and OLC numbers. Basically, the forced ectopic expression of transcription factors to direct ovarian and non-ovarian stem cells towards in vitro oogenesis has been utilized on various targets including exit from pluripotency, PGCLC development, the initiation of meiosis (haploid cell generation), OLC differentiation, OLC maturation, and, finally, the utilization of these female germ cells to produce viable and healthy offspring. Unfortunately, only a few researchers have succeeded in achieving these goals [33,59]. However, from time to time efforts have been made while using different sets of transcription factors and varied culturing conditions. To achieve such goals, researchers have targeted germ cell-specific transcription factors like PRDM1 (which is PR domain zinc finger protein 1 also known as BLIMP1), PR domain containing 14 (PRDM14), DAZL, Boule homolog, RNA binding protein (BOULE), BOLL (Boule like protein), factor in germline alpha (FIGLα) and FRAGILIS to differentiate stem cells into PGCs, OLCs, or follicular-like cells [71,73,74,75,76]. In a multi transcription factor study that targeted PGC differentiation, the Surani group tried the induction of three transcription factors, namely BLIMP1, PRDM14 and transcription factor AP-2 gamma (AP2γ) [71]. While checking the ectopic response of these three transcription factors (alone or collectively) on P19 embryonal carcinoma cells (P19EC) that originated from the mouse embryonic development day 7.5 (E7.5) epiblast, it was found that induction resulted in somatic genes repression, whereas PGC genes were induced. These results demonstrated that with the help of these factors, a PGC-like cell fate can be achieved even in the absence of cytokines. In accordance with such research outcomes, the simultaneous overexpression of these transcription factors (BLIMP1, PRDM14 and AP2γ) resulted in epiblast-like cell induction (EpiLCs) that could further form PGCLCs [73]. In another study, the ectopic expression of PRDM1/BLIMP1 in human ESCs promoted the generation of cells that resembled early PGCs, whereas the knockdown of PRDM1 resulted in impaired germline potential and upregulated neural-specific genes [74]. Mechanistically, it was observed that PRDM1 suppressed the transcription of sex determining region Y-box 2 (SOX2) and resulted in germline-specific lineage. While working with human ESCs, Irie and colleagues [75] demonstrated the association of BLIMP1 expression with SRY-box 17 (SOX17) and finally revealed SOX17 as a critical regulator of human PGCLC specification. It was speculated that BLIMP1, which represses the somatic genes, might allow SOX17 to regulate PGCLCs. However, unlike human germline cells, SOX17 is absent in mouse germline cells, which may be attributed to divergence in embryonic developmental stages among both species. Fragilis—an interferon inducible transmembrane protein—has also been associated with germ cell specification [76]. To verify the role of Fragilis in PGC fate determination, 129/SvEv mouse strain E7.0–E7.5 embryos were dissected and evaluated for tissue non-specific alkaline phosphatase (TNAP)-positive PGCs by using single cell complementary DNA analysis. A strong Fragilis expression in the PGC cluster region was found, and this persisted until the late bud stage, where it gradually faded. Cells that showed a higher expression of Fragilis were also shown to have a high expression of Stella, which implicates the role of both of the markers in PGC and early germ cell fate specification in mice. FIGLα, also known as folliculogenesis-specific basic helix–loop–helix, is an oocyte-specific transcription factor that regulates the expression of multiple genes that are involved in primordial follicle formation, oocyte differentiation and folliculogenesis. It also regulates the expression of zona pellucida genes. The mutation or knocking down of the FIGLα gene has been associated with accelerated oocyte loss, the lacking of zona pellucida genes (Zp1, Zp2 and Zp3), and POF [3,77,78]. In a gender-dependent study, human umbilical cord-derived MSCs (hUCMSCs) that were transfected with exogenous Figlα were shown to exhibit an increased expression of endogenous Figlα and other germ cell markers such as Dazl, Gdf9 and Zp2 [7]. Moreover, when these cells (Brdu-labelled) were transplanted into a female mouse kidney capsule, Brdu-positive VASA, DAZL and ZP2 cells were localized for up to two months. These results indicated that irrespective of gender, both sex-derived hUCMSCs had the ability to differentiate into OLCs. Additionally, with the ectopic expression of FIGLα, the extent of differentiation could be enhanced to a greater extent. Germ cell-specific differentiation has also been achieved by targeting another marker named DAZL. DAZL is a germ cell-specific RNA-binding protein that is expressed by the prenatal and postnatal germ cells of both sexes. This marker has been localized in both the nucleus and cytoplasm of fetal germ cells and in the cytoplasm of developing oocytes. It is associated with the regulation of MVH and SCP3 in meiotic cells, and it also acts as a key intrinsic meiosis initiation factor [79]. Both ESCs and SDSCs have been successfully differentiated into OLCs by inducing the ectopic expression of DAZL [28,42]. Induced cells not only highly expressed germ cell-specific markers but also formulated ovarian follicles and seminiferous tubule-like structures [42]. It was found that DAZL regulates early events of germ cell differentiation of mESCs through the suppression of Nanog. Interestingly, germ cell nuclear antigen (GCNA) was induced in the Nanog-negative cells. Additionally, the knocking down of the cells with small interfering ribonucleic acid (siRNA) transfection resulted in the reduced expression of germ cell-specific molecules including Prdm1, Stella and Mvh. These results demonstrated that DAZL is required for the expression of these markers. Similar results were shown by porcine fetal SDSCs, which were used to form PGLCs and OLCs under the ectopic expression of DAZL [28]. The enhanced expression of key germ cell markers such as Fragilis, Oct4, Stella, Vasa and meiosis genes including Scp3, Dmc1, Stra8 and Rec8 was observed from a DAZL-transduced group in comparison to non-transfected cells. Like mESCs, DAZL knockdown by siRNA resulted in the abrogated expression of germ cells and meiosis-related genes. These results indicated DAZL as a master gene that not only controls germ cell differentiation by regulating the expression of other key germ cell markers but also acts as a meiosis initiator by regulating meiosis-specific key genes.

Both DAZL and BOULE are members of the DAZ family and correspond to RNA-binding proteins [80]. Under in vivo conditions, DAZL regulates the proper development of male and female gametes by regulating the expression of germ cell-specific genes. Its deficiency has been associated with the reduced expression of genes such as Oct4, Mvh, Stella, and germ cell nuclear antigen (GCNA) in prenatal mouse embryos, which ultimately resulted in complications like impaired progression and meiotic arrest in postnatal mouse models [81]. Recently, transfection with DAZL and BOULE promoted the differentiation ability of hESCs into ovarian follicle-like cells [16]. A gradual decrease in the OCT4-positive cells and an increase in DAZL-positive cells was observed from human fetal ovaries that were collected at different gestational weeks (12 to 20 weeks). The same pattern of pluripotency marker downregulation was seen when hESCs were modulated with increased DAZL expression along with use of BMP4 and BMP8a, and vice versa, when DAZL-specific small hairpin ribonucleic acid (shRNA) was used. Additionally, luciferase activity revealed the regulation of VASA, SCP3 and cell division cycle 25A (CDC25A) proteins when DAZL and BOULE were overexpressed in hESCs. Human ESCs showed pluripotency exit, PGCLC development, and meiosis progression (as evaluated by PRDM9 and phosphorylated H2A histone family X protein (γH2AX) expression) when both DAZL and BOULE were overexpressed together after BMP treatment. Interestingly, differentiated hESCs undergo ovarian follicle formation when treated with the ovarian follicle inducers GDF9 and BMP15. A description of the transfection or ectopic expression-based OLC differentiation studies is given in Table 2.

## 4. Follicular Fluid: A Magical OLC Differentiation Inducer

In an ovarian follicle, the follicular antrum is filled with a fluid called follicular fluid (FF) that surrounds the ovum. Oocyte maturation and ovulation are controlled by the microenvironment that is provided by the FF, which has a significant role in fertilization and early embryo development [87,88]. A number of chemical constituents including hormones, proteins, sugars, anti-apoptotic factors, reactive oxygen species, cytokines, and growth factors are present in the FF, which directly or indirectly governs many important oocyte-specific processes [36]. Due to the presence of such vital constituents, different concentrations of FF derived from various species have been used as magical OLC differentiation inducers in many of the ovarian as well as non-ovarian stem cell sources that are derived from different organisms [7,8,11,13,14,25,29,30,31,35,89]. In the literature, FF alone or in combination with other growth factors and cytokines has been used to induce OLCs in vitro. Ovarian tissue as well as theca-derived porcine, bovine, and human stem cells, when cultured in FF alone [29], in combination with widely reported bone morphogenic proteins (BMPs) [31], or co-cultured with human granulosa cells [89] have been successfully differentiated into OLCs and exhibited positive expression for AP activity, primordial germ cell and early markers (Oct4, Prdm1, Prdm14, Stella, Vasa and Dazl), meiosis markers (Scp3, Dmc1), late oocyte-specific markers (Gdf9, Zp1, Zp2, Zp3) and folliculogenesis markers (FSHR) at different time intervals. Differentiated OLCs have shown gradual expansion in their diameter and revealed steroidogenic potential by secreting estrogen and, hence, mimicking natural oocytes. The culturing of Oct4 transfected porcine ovarian stromal cells with porcine FF not only differentiated into OLCs in vitro but also showed their tendency to form ovarian follicles in vivo [29]. All of these studies used 5% FF to differentiate OLCs in vitro. Recently, neonatal sources, i.e., umbilical cords [7,14], Wharton’s jelly [10,12] and amniotic fluid-derived MSCs [8,11] have been efficiently utilized in evaluating their OLC differentiation potential in vitro. In order to evaluate the gender-dependent potential of hUCMSCs into OLCs, both male and female hUCMSCs were induced with 20% cattle FF [7]. Male and female MSCs that were induced for 14 days resembled oocytes with their large round cell morphology, showed a positive expression of germ cells and meiosis-specific markers at both mRNA and protein levels, and also secreted higher levels of estradiol in comparison to their untreated control counterparts. Female MSCs showed comparatively higher efficiency for OLC differentiation in comparison to male MSCs. However, the overall percentage of in vitro induced germ-like cells was low, and cells could not reach maturity. Similar results were shown by Hu and colleagues, who developed OLCs from human first-trimester umbilical cord MSCs by using a differentiation medium consisted of 25% human FF, a follicular stimulating hormone, a luteinizing hormone, and estradiol [14]. Differentiated cells resembled PGC and OLC morphologies with varying diameters ranging from 50 to 120 µm on various time points and showed a higher expression of germ cell- and meiosis-specific markers than control cells. Additionally, some of the differentiated round cells were surrounded by small cells that resembled cumulus–oocyte complex (COC)-like cells and also showed the development of a zona pellucida-like protective layer surrounding differentiated oocytes. Researchers have also documented the generation of OLCs from another neonatal MSC source, i.e., amniotic fluid. In one such study, E-cadherin was shown to play a very important role in differentiating human amniotic fluid-derived MSCs into OLCs by culturing the MSCs in media that were supplemented with 10% bovine FF and β-mercaptoethanol [8]. In this study, E-cadherin^+^ human amniotic fluid MSCs (hAFMSCs) were shown to have a higher OLC differentiation potential than E-cadherin^−^ hAFMSCs. The knocking down of E-cadherin resulted in reduced DAZL, as well as other germ cell- and meiosis-specific marker expression, and hence demonstrated the utility of E-cadherin in this study. A high expression of DAZL in E-cadherin^+^ hAFMSCs was found to be major factor that resulted in an elevation in the expression of germ cell- and meiosis-specific markers when induced with FF. hAFSCs have also been utilized to derive primordial follicle- and oocyte-like cells when cultured with a 5% porcine, FF-containing medium [11]. Over induction for 10 days, the loosening of OLCs and granulosa-like cells (GCs) was seen from the culture dish. These cells when further cultured in differentiation medium for another 7–10 days, and the spontaneous development of 50–120 µm diameter OLCs with zona pellucida and COCs was seen. However, only 2% of the differentiated cells with a positive expression of oogenesis- and folliculogenesis-related markers were observed, which was quite low. However, this study lacked the assessment of in vitro maturation and fertilization potential of the differentiated OLCs. The presence of a high potency with characteristics of both embryonic as well as adult MSCs can be attributed to the above-mentioned neonatal MSC sources towards their germ cell-specific differentiation potential. OLCs have also been generated from human menstrual blood-derived endometrial MSCs [15]. Menstrual blood, which is generally regarded as a biological waste material, contains valuable cells that not only possess stemness characteristics but also possess germ cell differentiation potential when induced under specific culturing conditions. Similar to above-mentioned reports, menstrual blood-derived MSCs showed OLC differentiation potential when induced with a 20% human FF containing medium. In a time-dependent study, induced cells gradually attained oocyte-like morphologies, follicle stimulating hormone receptor (FSHR) and luteotropic hormone receptor (LHR) expression, and other meiosis- and germ cell-specific markers, and they also showed gonadotropin stimulated estrogen and progesterone secretion. Additionally, the development of granulosa and theca-like cells was also seen, which further assisted in the development of blastocyst-like structures. Skin-derived MSCs (SDSCs) represent another valuable cell source that has revolutionized the field of germ cell research due to these cells’ natural tendency towards germ cell differentiation [18,25,26]. These researchers performed a number of experiments concerning germ cell differentiation from SDSCs. Due to germ cell-specific intrinsic features, SDSCs (especially from the neonatal skin) from various organisms have been used to attain germ cell lineage. Both human and porcine fetal SDSCs, when cultured in a differentiation medium consisting of 5% porcine FF, have been shown to develop into germ-like cells [13,25]. With the progression in induction, a subpopulation of porcine fetal SDSCs expressed oocyte-specific markers such as Oct4, Gdf9b, Vasa and Dazl. When a subpopulation of these cells was further differentiated, they transformed into follicle-like aggregates that secreted estradiol and progesterone under gonadotropin stimulation, expanded their size, and also expressed other important oocyte markers including zona pellucida and the meiosis marker SCP3. Additionally, some of them gradually formed parthenogenetic embryo-like structures [25]. On the other hand, consistent with these results, human fetal SDSCs were also successfully differentiated into early germ cells when induced under culturing conditions containing 5% porcine FF-conditioned media [13]. Interestingly, the evaluation of a punctate and elongated SCP3 staining pattern was observed from differentiated cells, which indicated the initiation of meiosis, a major obstacle to overcome during germ cell-specific differentiation in vitro. Recently, a new approach focusing on the use of demethylating agent 5-azacytidine (5-aza) was implemented to derive OLCs from cow skin fibroblasts [30]. In a dose- and time-dependent manner, skin fibroblasts, when treated with 2.0 µM of 5-aza for 72 h, not only changed their morphology from the fibroblast to the oval or round shape and remained viable to greater extent but also elevated their pluripotency marker (Oct4, Sox2, Nanog, Rex) expression. Furthermore, some populations of 5-aza treated cells, when treated with BMP2, BMP4 or 5% FF (7–14 days), attained an oocyte-like morphology with a large round shape and an enhanced diameter. Induction with BMP2, BMP4 or 5% FF showed a different but elevated pattern of germ cell- and meiosis-specific markers. Considering cell viability and response under differentiation inducers (e.g., chemicals, cytokines, and growth factors), other relevant agents such as sodium butyrate, valproic acid, and trichostatin-A can also be used in case of OLC differentiation. Interestingly, human FF-derived MSCs have also been utilized in generating OLCs under BMP15 induction (unpublished work) [90]. This study demonstrated the innate ability of FF-derived cells to possess stemness properties (fibroblastoid morphology, pluripotency marker expression, adipogenesis, and osteogenesis), the development of round OLC-like structures (with 20–30 µm size), and high estradiol secretion by freshly seeded cells followed by gradually decreased levels and positive expression of zona pellucida markers, i.e., ZP2 and ZP3. Under induction in a differentiation medium that was supplemented with BMP15, the size of the round OLCs was increased up to 115 µm. However, with the initial higher levels of estradiol and pluripotency and oocyte-specific marker expression in untreated cells than in BMP15-treated cells, a gradual downregulation was seen in both the cell groups. These results indicate that FF can also be the choice source among all above-mentioned sources when targeting OLC differentiation. 

## 5. Skin-Derived Stem Cells

Skin comprises a pool of many valuable stem cell types that are located in its epidermis, dermis, and hair follicles, and it has also the ability to continuously self-renew most of its tissues [91]. Considering these stemness properties, many researchers have reported the production of primordial germ-like cells (PGCLCs), germ cell-like cells (GCLCs) and, more specifically, oocyte-like cells (OLCs) from mouse [19,24,37], porcine [25,26,28,92], bovine [30] and human [13] SDSCs. These reports demonstrated that fetal SDSCs from mice, porcine, and humans, when induced under appropriate conditions (transfection, embryoid body (EB) formation, spheroid formation, use of growth factors like BMPs, demethylating agents like 5-aza, and culturing in FF containing media), can be differentiated into female GCLCs. As stated by Dyce and colleagues, the ability of SDSCs to develop into GCLCs in vitro can be attributed to the possibility of the presence of germ cell-specific intrinsic properties in the somatic cells (fetal skin cells) from the later fetal developmental stages. Keeping this idea in mind, germline cells were produced from porcine fetal SDSCs [25]. Fetal porcine SDSCs, when cultured in 5% porcine FF containing media for about 40 days, developed into GCLCs with the expression of Oct4, Gdf9b, Dazl and Vasa and, in a suspension culture, formed spherical aggregates that resembled COC-like structures. Furthermore, large OLCs, which showed positive expression for late female germ cell markers like ZP and SCP3 and also spontaneously developed into parthenogenetic embryo-like structures, were extruded from these aggregates. Under natural conditions, primordial germ cell (PGC) development occurs prior to germ cells or oocytes. To mimic same developmental process in vitro, Linher and colleagues utilized fetal porcine SDSCs and showed their ability to form germ cell precursors, i.e., PGCLCs [92]. When distinct non-adherent spheroid-like skin clusters were cultured in a 5% porcine FF containing medium, around 1.4% of the total seeded cells transformed into round PGCLCs and exhibited 15-fold higher AP activity than undifferentiated cells. RT-PCR and immunostaining results also revealed higher levels of PGC markers such as OCT4, FRAGILIS, STELLA and DAZL in induced cells. More importantly, sodium bisulfite sequencing revealed imprint erasure by induced PGCLCs, which is a characteristic feature of naturally developing PGCs in vivo. Additionally, induced PGCLCs transduced with a DAZL-GFP promoter could be further developed into OLCs but with very low cell population. To evaluate the in vitro oogenesis to further extent, the same group of researchers, along with others, again developed OLCs from both male and female porcine fetus-derived SDSCs and demonstrated the expression of cell adhesion and gap junction proteins connexin37 (CX37) and connexin43 (CX43). Like in vivo derived oocytes, chromosomal spreads from very small population of differentiated cells showed similar patterns of nuclei as being present during different stages (leptotene, zygotene and pachytene) of the prophase-1 of meiosis. Differentiated cells also revealed similar demethylation patterns to naïve oocytes [26]. DAZL overexpression also assisted in the formation of PGCLCs and OLCs from porcine SDSCs [28]. DAZL-transduced cells showed an enhanced expression of germ cell markers such as Oct4, Stella and Vasa, which was suppressed with the introduction of DAZL-specific siRNA. The knockdown of DAZL also resulted in the downregulation of meiosis-related genes such as Scp3, Dmc1, Stra8 and Rec1, whose expression was elevated during the later stages of differentiation (under DAZL overexpression). These results indicated that the introduction of exogenous markers can be used as an alternative method to induce GCLC differentiation from somatic and stem cells.

One other approach to derive PGCLCs and GCLCs from SDSCs could be the use of cytokines, chemicals, or small molecules. In support of this hypothesis, mouse SDSCs have been transformed by using Activin-A, 5-aza and RA with or without induction of BMPs and FF in the differentiation medium [21,24,30,37]. Under natural conditions, Activin-A, a member of the TGF-β superfamily, promotes oogenesis and folliculogenesis. In a report by Sun and colleagues, GFP transgenic mouse SDSCs were transformed into PGCLCs through embryoid body-like structure (EBLS) formation with or without Activin-A followed by co-culturing with mouse embryonic fibroblast (MEF) cells in a differentiation medium that was supplemented with or without Activin-A [37]. The co-culturing of EBLSs with MEF cells for 15 days enhanced the expression of both early (Fragilis and Stella) and late (Figla, Nobox and Dazl) PGC markers. Moreover, the percentage of SSEA-1 and GFP double positive PGCLCs was increased in 0–100 ng/mL Activin-A induced EBLSs, which upon further analysis, showed similar epigenetic modifications (reduced cytosine methylation (5Mc) and elevated levels of H3K27me3 and 5-hydroxymethylcytosine (5hmC)) to in vivo formed PGCs. The interruption of stimulatory effect of Activin A by a Smad3 RNA interference (RNAi) assay revealed that SMAD3 acted as a key effector molecule in regulating such processes by the Activin-A signaling pathway. Interestingly, a reduced percentage of PGCLCs whereas increased expression of meiosis-specific genes such as Stra8, Sycp1, Sycp3 and Dmc1 was seen at 16th day of differentiation, which indicated that Activin-A not only promoted PGCLC differentiation from SDSCs during early stages but also modulated meiotic initiation in PGCLCs during later stages. Like porcine, mouse, and bovine SDSCs, human SDSCs also have the ability to differentiate into OLCs when induced under appropriate culturing conditions. In a recent report by Ge and colleagues [13], human fetus SDSCs, when induced under appropriate conditions in a medium containing 5% porcine FF, were differentiated into early GCLCs without the integration of any exogenous gene. In vitro cultured human SDSCs showed higher levels of pluripotency markers (Oct4, Sox2 and Nanog), had a stable karyotype, and formed EBs with the positive expression of STELLA and VASA. When human SDSCs were differentiated into porcine FF supplemented media, vesicular structures were formed, and these showed elevated levels of germ cell (Dazl, Vasa), meiosis (Scp3) and granulosa cell (Amh) markers. Interestingly, after 25 days of differentiation, some of the differentiated cells showed 1.79% of putative haploid cells (1N) population (evaluated by FACS) and also exhibited punctate and elongated staining patterns (evaluated by immunostaining), which indicated meiotic progression and the future possibility to develop into both male and female GCLCs. However, when using this protocol, elevated levels of Amh in differentiated vesicular cells led researchers to speculate that they may develop into female GCLCs when induced under appropriate conditions. However, in this study, the researcher could not differentiate human SDSCs into OLCs and suggested more optimized differentiation conditions including the use of human FF (if possible). Male and female gamete development has also been regulated by the RA signaling pathway, which not only assists in PGC growth, meiosis initiation, and the derivation of competent oocytes from PGCs but also promotes PGCLC proliferation and germ cell development from mouse SDSCs [21,24]. In a recent study, the differentiation of PGCLCs from mouse SDSCs was mediated through EB formation followed by co-culturing with mouse fibroblast cells. Induction with different concentration of RA resulted in PGCLC proliferation, which was confirmed by a higher ratio of 5-bromo-2-deoxyuridine-positive PGCLCs (immunofluorescence), an elevation in the number of cells in the S-phase of cell cycle (FACS), and increased mRNA levels of cell cycle-related genes such as Ccnd1 and Cdk2. Moreover, the endogenous PGC proliferation rate was increased when fetal gonad genital ridges were treated with 5 µM RA in vitro [21]. In a similar study, newborn transgenic mice-derived SDSCs were differentiated into GCLCs under RA treatment. A significant elevation in the mRNA levels of meiosis marker genes (Stra8 and Sycp3), a meiosis regulatory gene (Marf1), and an oocyte marker (Oct4), a reduced expression of a meiosis inhibitory gene (Nanos2), and an increased expression of gap junction and pluripotency marker proteins CX43 and OCT4 confirmed the meiosis initiation and GCLC differentiation. Additionally, the elevated expression and improved localization of oocyte-specific markers SCP3 and ZP3 further confirmed the successful differentiation and structural integrity of the OLCs [24]. From the overall results, it can be formulated that intrinsic properties for GCLC differentiation from SDSCs can be promoted by induction with appropriate culturing conditions, with a special focus on the use of TGF-β family-related cytokines (Activin-A and BMPs), vitamin-A derivative (RA), demethylating agents (5-aza), co-culturing with MEF, and by adding appropriate concentrations of FF. By keeping cellular survivability, safety, toxicity and the extent of differentiation potential in mind, some other suitable and novel differentiation inducers should also be developed.

## 6. Co-Culturing as an Efficient Method

In developmental biology, the generation and proper functioning of any tissue or organ is associated with the efficiency of the concerned cell, as well as its surrounding environment (cellular niche). Cells exhibit autocrine and paracrine signals that directly or indirectly activate or downregulate many important pathways and ultimately lead to the development, transformation, maturation, or proper functioning of the cells and concerned tissues. In cytological studies, co-culturing system can be highly useful to transform many cell lineages. Many researchers have utilized this phenomenon to differentiate various sources-derived stem cells to develop PGCLCs or GCLCs by using MEF co-culture [37,41], granulosa cell co-culture [15,17,22,84,86], placental cell co-culture [12], ovarian stem cell co-culture [20], and fetal gonadal cells co-culture [82]. During whole developmental stages of oogenesis, a developing oocyte is surrounded by different cell layers whose number and shape spontaneously change at different intervals of time. Examples of such gradually developing and surrounding cells are pre-granulosa cells, granulosa cells, and theca cells. Granulosa cells produces the key steroid hormone estradiol (E2), whose synthesis is based on the collaborative relationship between theca and granulosa cells. Androgens such as dehydroepiandrosterone (DHEA), androstenediol, androstenedione, and testosterone produced by theca cells in response to luteinizing hormone (LH) are diffused into granulosa cells and are further converted to estrogens (estrone and estradiol) in granulosa cells via the enzyme cytochrome P450 aromatase (CYP19A1) [93]. These cells (granulosa and theca cells) regulate in vivo oogenesis by secreting autocrine and paracrine signals. Therefore, to differentiate stem cells into PGCLCs, GCLCs or OLCS, many researchers have adopted the co-culturing of granulosa cells, either directly (cell inactivation) or indirectly (transwell co-culture system). In a two-step method, mouse ESCs were firstly differentiated into PGCs through EB formation (four days), and then these EBs were co-cultured with ovarian granulosa cells (another 10 days) to obtain OLCs [17]. Induced cells (EBs co-cultured with granulosa cells) showed a positive expression of oocyte-specific markers such as Figlα, Gdf9 and zona pellucida genes (Zp1-3). However no testis-specific gene expression was observed in the differentiated cells. Positive immunocytochemical expression for Mvh and GDF9 markers further confirmed successful differentiation. Interestingly, none of the conditions—neither EB culturing alone or with a granulosa cell-conditioned medium nor EB cell co-culturing with Chinese hamster ovary (CHO) cells or with a CHO cell conditioned medium—could result in the expression of oocyte-specific markers. These results indicated the efficacy of PGC and granulosa cell interactions to develop OLCs. More recently, in a stepwise manner, mouse ESCs were utilized to derive EBs under BMP induction and further into OLCs by treating the EBs with RA and co-culturing with mitomycinized OSCs [84]. Mouse ESCs that were induced to EBs by using BMP4 resulted in a significant increase in the expression of meiotic markers (Stra8, Rec8, Scp1 and Scp3) in comparison to both BMP4-lacking and monolayer cell culturing conditions. Furthermore, BMP4-induced EBs, when treated with RA and co-cultured with ovarian somatic cells for up to 14 days, exhibited round oocyte-like cell morphology and showed significantly higher levels of germ cell (Mvh) and maturation markers (Cx37, Zp2, and Gdf9). However, RA treatment resulted in a significant decline of Oct4 expression (both at mRNA and protein levels) when compared to control cells. Just like ESCs, both human and murine-derived OSCs have also been shown to enhance OLC differentiation potential when co-cultured with granulosa cells [22,86,89]. By using a morphology-based selection method, isolated murine OSC colonies were transferred onto inactivated granulosa cells to evaluate their ability to differentiate into OLCs [22]. Despite having positive expressions of Oct4, Nanog, C-kit, Fragilis, Dazl and Mvh, OSC colonies lacked the expression for meiosis- (Scp3) and oocyte-specific marker (Gdf9). OSC colonies were found positive for AP activity and also showed positive immunofluorescence results for OCT4, SSEA1, DAZL, and MVH markers. Furthermore, co-culturing with granulosa cells for up to 11 days resulted in the evaluation of SCP3 and GDF9 markers both at mRNA and protein levels that were absent earlier in normal OSCs. However, there was no change in the overall appearance and propagation rate of the cells, which indicated their early developmental stage, like mouse ESC-derived OLCs [17]. In similar research by Zou and colleagues [86], murine female germline stem cells (FGSCs), when induced under five different conditions, showed higher differentiation potential towards germinal vesicle (GV) stage oocytes under a hanging drop procedure and co-culturing with inactivated granulosa cells, among all other methods. Consistent with the Parvari et al. [22], FGSCs lacked meiosis-specific marker expression despite having expression for other germ cell markers. However, in comparison to other differentiation strategies, induction under differentiation conditions (hanging drop procedure and granulosa cell co-culture) for up to 18 days gradually enhanced the cells’ diameters (more than 50 µm) and developed structures like GV and zona pellucida and, hence, confirmed the induction condition to be optimal. Moreover, an enlarged diameter and chromatin nuclear proportion, nucleus–plasma ratio of GV oocytes differentiated from murine FGSC-like GV oocytes (in vivo), compared to undifferentiated FGSCs (as evaluated by three dimensional (3D)-reconstructed images), indicated a successful differentiation into GV-stage oocytes. Consistent with the mouse ESCs, OSCs, and FGSCs, human ovarian theca-derived stem cells (hTSCs) have also shown the ability to differentiate into OLCs when co-cultured with granulosa cells [89]. In this study, hTSCs were induced with a 50-day-long differentiation protocol that utilized a 5% human FF supplemented differentiation medium. With the extension of induction time, the 25-day-treated cells became round-sized (20–25 µm diameter) and, when further co-cultured with human granulosa cells for another one week, enlarged their diameters up to 60–70 µm and resembled OLCs. Differentiated OLCs were found positive for Oct4, Prdm1, Prdm14, Vasa and Dazl (PGC and germ cell-specific genes) as well as Gdf9, Zp1, Zp2 and Zp3 (oocyte-specific genes) and meiosis-specific markers (Dmc1 and Scp3). Additionally, the localization of OCT4, DAZL, VASA, GDF9 and ZP3 proteins was also observed in differentiated OLCs. These observations have provide researchers deep insights for using theca cells regarding OLC differentiation. Stem cells from non-ovarian sources have also shown the ability to differentiate into OLCs under granulosa cell co-culturing [15]. All of these studies have demonstrated that granulosa cells transduce multiple signals, which assists in meiosis initiation and oocyte maturation through cell-to-cell contacts (contact between induced and granulosa cells) via gap junction and paracrine mechanisms. However, morphological improvements (shape and size), the expression of all PGCs, meiosis- and oocyte-specific markers, a lack of apoptosis, and, above all, an in vivo functional ability of the differentiated cells to produce viable offspring are most important and un-negotiable, as none of the above-mentioned studies could create viable offspring. Therefore, much more core studies will be needed to make these OLCs an errorless tool to treat female reproductive disorders. A complete description of the co-culturing-based OLC differentiation studies is given in Table 3.

## 7. Very Small Embryonic-Like Stem Cells (VSELS): New Heroes in Gamete Research

**Figure 1:**
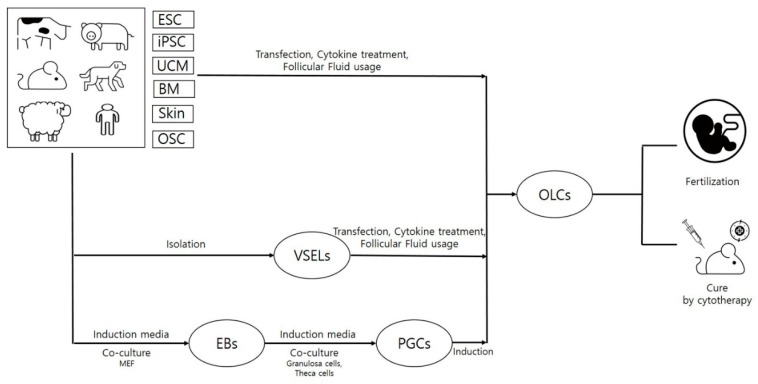
Schematic presentation of stem cell-derived oocyte-like cells when using various strategies and their in vivo efficacies. This figure demonstrates that stem cells (embryonic stem cells (ESCs), induced pluripotent stem cells (iPSCs), and mesenchymal stem cells (MSCs)) can be obtained from various organs of different organisms. These cells, upon treatment with specific cytokines, growth factors or differentiation inducers, can be used to differentiate oocyte-like cells (OLCs). These differentiated cells can be further transplanted to disease models to cure the disease.

ESCs, iPSCs and MSCs (neonatal and adult) constitute the main types of stem cells. However, there is another type of very small-sized (3–7 μm) cells that are called very small embryonic-like stem cells (VSELs) and have stem cell-like characteristics, and, due to their high potency, they can be used in regenerative therapy as alternative cell sources against ESCs and iPSCs [97]. Due to their very small size, special gating strategies are required during their isolation through FACS. Characteristic similarity with early developmental cells, ESCs-like behavior (SSEA, Oct4A, Nanog and Rex1 expression), relevance with PGCs (Stella and Fragilis expression) and gonads (isolation from OSE as well as testes), and ex vivo proliferation ability without using feeder layers or any viral transductions makes VSELs a highly valuable cell source for regenerative medicine. According to a recent report by Lahlil and colleagues [98], VSELs not only showed expansion but also retained their pluripotency and differentiation abilities when cultured under feeder free conditions and using specific media (supplemented with a pyrimidoindole derivative UM171). Moreover, resistance to growth factors such as insulin or insulin-like growth factors and changes in parentally imprinted gene expression regulate VSELs’ quiescent state in adult tissues. More importantly, under conditions like organ damage, activated VSELs can contribute to the regeneration of damaged organ tissues by their mobilization in peripheral blood [99]. Due to their detection in PGCs, gonadal tissue (both functional and non-functional), and ability to survive oncotherapy (due to quiescent nature), VSELs have been given preference for the development of gametes in vitro [45,100]. Adult mammalian ovaries not only acts as reservoirs for oocytes but also have the presence of other valuable stem cell sources such as OSCs and VSELs. Despite having smaller sizes than OSCs, VSELs express nuclear OCT4 rather than cytoplasmic OCT4 in OSCs, which defines their pluripotent nature [101]. These pluripotent cells can be isolated from a number of other adult cell sources such as bone marrow [102], bone [103], umbilical cord blood [104], and peripheral blood [105], and they can be used to derive OLCs when induced under suitable culturing conditions. Due to the quiescent nature of VSELs, they can avoid nuclear and mitochondrial mutations and can therefore be used as safer therapeutic agents in reproductive medicine [106]. However, whether VSEL-derived OLCs can behave like fully functional oocytes is still a big mystery. To address this question, Virant-Klun and colleagues carried out one study to check the functional ability of VSEL-derived OLCs to get fertilized in the presence of sperm. Interestingly, OLCs could recognize the sperm and released zona pellucida-like structures, but they could not undergo fertilization, demonstrating their immature nature and functional incompetency [107]. Therefore, more research is still needed to achieve fully functional OLCs. A schematic representation of OLC differentiation strategies and their use in curing diseased animal models is given in Figure 1.

## 8. Direct Utilization of Undifferentiated Stem Cells in Curing Fertility Disorders

**Figure 2:**
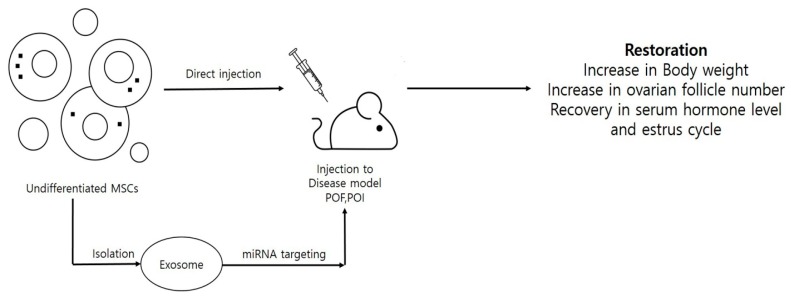
Stem cell utilization for restoration of reproductive properties in diseased animal models. This figure demonstrates that stem cells can be directly injected to cure the diseased animals either by stem cells induced paracrine signaling or by secreted microRNA (miRNAs)/very small embryonic-like stem cells (VSELs).

Therapeutic applications (like chemo or radiation therapy) results in reproductive damage and cause pathological conditions like premature ovarian failure (POF), premature ovarian insufficiency (POI), or infertility by inducing dysfunction, depletion and cytotoxicity in reproductive structures including follicles, oocytes, and granulosa cells. Moreover, symptoms like hormonal imbalance, reduced follicle numbers, and granulosa cell apoptosis have been commonly seen in the patients suffering from POF or POI. To study disease pathology and preventive measures, a number of POF and POI disease models have been developed, either by injecting specific doses of cytotoxic chemicals like cisplatin, cyclophosphamide and busulfan into animal models or by harming ovaries with certain agents like hydrogen peroxide (H_2_O_2_). The use of stem cells in curing female reproductive disorders is depicted in Figure 2. One alternative approach to overcome such problems can be the use of stem cells as cellular therapeutic agents. Multilineage differentiation potential, high proliferation rates, and immunomodulatory properties have made stem cells a booming cell source in the field of regenerative medicine. Despite having the ability to differentiate into many other cell types under targeted lineage-specific culture conditions, stem cells have shown promising results in curing diseases by their direct insertion into disease models. A number of neonatal and adult sources including bone marrow [43,47,48,55,108,109], endometrial [110], amnion [53,111], amniotic fluid [4], amniotic epithelium [44], menstrual blood [9,51], umbilical cord [56,112], umbilical cord blood [54], umbilical cord vein [52], placenta [49], chorionic plate [50], adipose [113], and skin [46] have shown the restoration of ovarian function in many reproductive disease models. Due to their low immunogenicity, migratory, and homing properties, MSCs have been widely used to restore ovarian functions in POF models. Estrus cycle recovery, reduced apoptosis rates in granulosa cells, elevated estradiol levels (E_2_), increases in body weight and ovarian index, a healthy status of the follicles with increment in their counts (neo-folliculogenesis), and, finally, the ability to undergo fertilization and attain fertility are some of the post-transplantation features that have been reported in various POF and POI animal models [4,9,47,48,108,110]. In a recent report, the ovarian function in a chemically-induced POI mouse model was recovered by using human menstrual blood-derived MSCs (MenSCs). Bioinformatics analysis and mRNA sequencing results revealed that the activation of ECM-dependent focal adhesion kinase/serine-threonine protein kinase (FAK/AKT) signaling pathway by MenSCs was associated with the restoration of ovarian function [51]. In another report, the administration of CD44^+^ and CD105^+^ hAFSCs was shown to survive and proliferate in a chemotherapy-induced POF mouse model. The presence of red fluorescent protein-labeled hAFSCs was observed along the injection tract in POF mouse ovaries, even after three weeks [4]. More recently, a review of literature discussed the presence of stemness characteristics and OLC differentiation abilities of ovarian cortex-derived OSCs [114]. The authors advocated the developmental ability of both the non-menopausal and menopausal women-derived OSCs to undergo in vitro differentiation into mature OLCs and strongly emphasized OSC-derived OLCs use in treating the ovarian insufficiencies. Additionally, it was stated that DDX4^+^ OSCs, when cultured with FSH and EGF, can acquire mesenchymal characteristics, e.g., stem cell plasticity and pluripotency marker expression. However, the authors also raised concerns about the possibility of DDX4^+^ OSCs in contributing carcinogenesis during ovarian ageing. Therefore, the enrichment of mesenchymal markers and long term in vivo survivability should also be considered during MSC selection for treating such disorders. Along with mesenchymal characteristics, culturing methods should also be given importance when selecting and preparing MSCs for certain disease models. To support this hypothesis, Kim and colleagues [49] demonstrated that placenta-derived MSCs, when cultured under 3D conditions and changed to spheroid form, can enhance ovarian function by inducing folliculogenesis in ovariectomized rat models. In comparison to conventional monolayer (two dimensional: 2D) cultured and control groups, transplanted 3D spheroids not only showed enhanced engraftment efficiency and E_2_ levels but also showed the increased expression of folliculogenesis-related markers such as Nanos3, Nobox, and Lhx8. Recently, researchers have used MSC-derived exosomes or microvesicles to evaluate their effect on diseased models [43,44,45]. In a chemotherapy-induced rat ovarian failure study, the transplantation of bone marrow MSCs (BMSCs)-derived exosomes exhibited an increase in the number of basal and sinus follicles, elevated levels of E2 and AMH and reduced levels of FSH and LH in the serum, and it also improved the estrus cycle in rat POF models [55]. The main reason behind these improvements was found to be episomal micro RNA, i.e., miR-144-5p derived from BMSCs exosomes. Upon co-culturing cyclophosphamide damaged granulosa cells with BMSC exosomes, BMSCs derived exosomal miR-144-5p was found to downregulate phosphatase and tensin homolog (PTEN) protein expression via inactivating the phosphoinositide-3-kinase/serine-threonine protein kinase (PI3K/AKT) pathway, whose PTEN expression was high in apoptotic granulosa cells. In a similar way, human amniotic epithelial cell (hAEC)-derived exosomal microRNA, i.e., miR-1246, played a key role in restoring ovarian function in a chemotherapy-induced mice POF model [44]. In this study, hAEC-derived exosomes inhibited granulosa cell apoptosis, protected ovarian vasculature, and maintained the number of primordial follicles in the chemotherapy-damaged ovaries. Similar results were shown by Sun and colleagues [43] who demonstrated the inhibition of ovarian granulosa cells apoptosis by negatively regulating p53 by the administration of BMSC-derived exosomal miR-644-5p into a cisplatin-induced POF mouse model. These results indicated that MSC-derived exosomal miRNA can play a key role in the restoration of ovarian function in chemotherapy-induced animal models. A complete description MSCs derived from different sources and their therapeutic potential to cure reproductive disease animal models is given in Table 4. Due to vast advancements in research methodologies and valuable outputs, the stem cell field has not only restricted to in vitro transdifferentiation and cell engineering but has also been given high consideration in ongoing clinical trials. A number of clinical trials are being conducted to deal with the female reproductive disorders and are expected to deliver desired and safe results (https://stemcellsportal.com/clinical_trials_infertility). A detailed description is given in Table 5.

## 9. In Vivo Efficacy of the In Vitro Differentiated OLCs

Ovaries are the richest source of functional oocytes and also act as reservoirs to different cell sources including OSCs, GCs, COCs and VSELs. Ovaries undergo a series of gradual developmental events before producing fully functional (fertilizable) oocytes. However, under in vitro conditions, it is very hard to generate functional OLCs due to the absence of favorable developmental conditions and surrounding niches. Researchers from various laboratories have tried to generate OLCs, but their functional competence to produce viable and healthy offspring has remained a far sighted task for most of them. This difficult task of getting functional OLCs could be made possible with use of ESCs, embryonic PGCs or oogonial cells, and ovarian FGSCs [33,59,116,117,118,119]. Among all such studies, the majority of the success has been associated with the use of ovarian fragments, reconstituted ovaries, fetal PGCs or oogonial cells [116,117], and FGSCs [33,59,119]. After isolation, these cells were manipulated under in vitro culturing conditions and finally transplanted to the partially or whole ovariectomized mouse models to check their efficiency to produce viable offspring. In a series of different experimental set ups to obtain offspring, the Nakatsuji group tried various strategies including the isolation of ovaries (ovarian fragments or 12.5–14.5 days post coitum [dpc] intact fetal ovaries) or ovarian cells (oogonial and fetal oocytes), their culturing (organ or dissociate culture system), and in vitro manipulation [116]. Upon transplantation into ovariectomized mouse models, viable offspring were produced. In a different study, PGCs that were collected from 12.5 dpc male and female gonads along with their gonadal somatic cells were reaggregated, filtered, and cultured for up to 18 h and finally transplanted to the bilaterally ovariectomized same strain recipient mice. Interestingly, after four weeks, transplanted cells became round spermatids or germinal vesicle (GV) stage oocytes and produced normal pups when injected with in vivo derived oocytes and normal spermatozoa, respectively [117]. These results indicated the possibilities of developing fertilizable germ cells from embryonic state stem cells where developmental efficiency can be further improved by using specific supportive cells (somatic cells) like gonadal somatic cells. By using similar approach, neonatal ovary-derived FGSCs were maintained for a longer duration and also produced viable offspring when transplanted into infertile mouse ovaries [33]. Furthermore, the role of gene manipulation (using viral infection) in FGSCs that were derived from both the neonatal and adult FGSCs was evaluated to produce transgenic mice [59]. Both the neonatal and adult ovary-derived FGSCs were in vitro cultured for short duration (three-to-five days) and virus particles infected with targeted genes *MSCV-PGK-GFP* (carrying GFP protein). These infected FGSCs, when transplanted into chemo ablated recipient mouse ovaries, not only showed the presence of GFP-positive oocytes (after two months post transplantation) but also demonstrated a high efficiency for fertility restoration by producing viable and healthy offspring. FGSCs were further evaluated to understand the role of another targeted genes such as Oocyte-G1 and Dnaic2. These studies indicated the utility of FGSC-mediated transgenesis to produce transgenic offspring. Though there have always been some concerns regarding the reliability of the methods used to isolate FGSCs, researchers have tried to address these concerns by developing safer and more efficient isolation procedures. Recently, Shixuan Wang lab developed a new differential adhesion method to isolate FGSCs from mouse ovaries [119]. Isolated FGSCs showed a spontaneous in vitro OLC differentiation ability, as evaluated by a number of germ cell-, meiotic- and oocyte-specific genes including Mvh, Tert, Fragilis, Dazl, Dppa3, Prdm1, Scp3, Dmc1, Ybx2, Zp3 and Gdf9, and they also exhibited stable karyotype and AP activity. FACS-based ploidy analysis revealed the presence of 3.16% cells with the haploid status. Furthermore, the transplantation of these FGSC-derived OLCs into recipient female ovaries resulted in healthy offspring production with very high efficiency (86%). The overall results of this study found that the phosphoinositide-3 kinase (PI3K)-AKT pathway was responsible for regulating FGSCs’ self-renewability. Considering the safety and reliability of the isolation protocols and also developing methodologies to get high yield after in vitro propagation, FGSCs can be used as tools to cure infertility. Researchers have also succeeded in producing viable offspring by using pluripotent stem cell types rather than by using ovary-derived PGCs or FGSCs. This was made possible by using ESCs and iPSC-derived OLCs. In a stepwise differentiation process, Hayashi and colleagues [118] firstly developed PGCLCs from mouse ESCs and iPSCs, which, upon aggregation with female gonadal somatic cells, produced reconstituted ovaries. Finally, when these aggregates were transplanted under ovarian bursa, fertile offspring were produced. This groundbreaking study laid a guiding milestone in reproductive biology research field and indicated that the selection of suitable cell source (pluripotency status), treatment with appropriate differentiation inducers and assistance with suitable and supportive cell niche constitute the basis for the development of functional OLCs. In a very interesting study, the laparoscopic injection of autologous BMSCs (derived from iliac crest) into the ovaries of POF patients resulted in fascinating results. Out of 10 POF patients, two patients resumed menstruation and one patient showed improved ovarian reserve score, attained pregnancy, and delivered a full term healthy baby [115]. Therefore, stem cells not only differentiate into OLCs under specific in vitro conditions but also have the ability to cure the reproductive diseases by secreting autocrine and paracrine signals when transplanted directly into the body. 

## 10. Selection of Best OLC Source for Fertility Treatment

From all the above-discussed available cell sources with the ability to undergo OLC differentiation, it has been observed that ovary-derived cells (ovarian cortex, OSE) and VSELs are the best cell sources due to their advanced features. Due to the high pluripotency of ESCs and iPSCs, these cells can be converted to any cell type, but ethical issues and safety (teratoma formation) concerns restrict their usage, especially for germ cell differentiation (which are the basis of new life). Additionally, neonatal and adult MSCs are less pluripotent and have shown the development of immature oocytes even when induced under complex and very expensive culture conditions. Moreover, no ESCs, iPSCs and MSCs have shown their ability to withhold cancer treatments. On the other hand, ovarian tissue-derived stem cells have a natural tendency for OLC differentiation. OSCs can be easily differentiated to OLCs under minimal culturing conditions. These cells can also be stored earlier in the case of patients undergoing cancer therapies. As far as germ cell specificity is concerned, VSELs hold the same importance like OSCs. These small-sized cells possess ESC-like characteristics, PGC marker expression, and the ability to be isolated from gonads. Most importantly, they can survive oncotherapy and can therefore be considered the best sources for germ cell differentiation and fertility restoration. However, complex VSELs isolation protocols, the low availability of ovarian tissues, and a dependency on autologous stem cell sources (to avoid immune rejection) seem to be obstacles for their global use for fertility treatments. Therefore, there is an urgent need for refinement in the available strategies so that stem cells can be easily isolated, safely propagated, and effectively differentiated to any target lineages without causing any safety issues.

## 11. Present Complications and Future Expectations

In comparison to MSCs, ESCs and iPSCs have shown a greater ability towards OLC differentiation. The pluripotent nature of these stem cell types is the main reason behind such desired outcomes. Successful in vitro OLC differentiation requires a series of gradual developmental events including exit from pluripotency, morphological alteration (round oocyte-like cell morphology), early and late PGC maker expression, meiosis entry, oocyte-specific marker expression, the development of a protective zona pellucida layer as well as nourishing granulosa and theca cell layers, cumulus–oocyte complex formation, and cell size expansion at regular intervals. During differentiation, the majority of the cell types attain early marker profiling, but typical oocyte-like morphology, meiosis initiation, and the expression of post-meiotic and mature oocyte markers seem to be the major obstacles. The non-expansion of cell size and the lacking of zona pellucida, granulosa and theca layers further leads to the development of immature oocytes. Moreover, the use of ESCs or iPSCs may result in the derivation of functional OLCs, but ethical concerns and safety issues may contribute to their negligence. Therefore, more attention needs to be paid to eliminate these issues, or highly efficient protocols must be developed to get similar results by using other non-ethical and safer stem cells sources. During the selection of a candidate stem cell source, stable long term expansion, low immunogenicity, donor age and the screening of certain OLC promoting factors like E-cadherin [8] and its pluripotency marker status must be considered at a higher priority. Neonatal sources like placenta, the umbilical cord (UC), umbilical cord blood (UCB), and Wharton’s jelly (WJ), which have been proven to be more potent and equipped with ESC-like characteristics, must be given importance. Follicular fluid, which restores many vital elements for oocyte growth in vivo and OLC differentiation in vitro, should be analyzed in depth to find out the number and composition of such vital elements so that they can be proved helpful during the development of highly efficient protocols and dependency of most widely used follicular fluid can be eliminated. 

## 12. Conclusions

On the basis of various reports and research outputs (merits and demerits), it has been concluded that stem cells not only have the potency to differentiate into OLCs but can also cure female reproductive disorders after being transplanted into disease models (in their induced or non-induced form). However, many more protocol refinements are still needed to make fully functional OLCs. Additionally, ovarian tissue-derived cells (OSCs) and VSELs can be the best choices of cell sources for fertility treatment. These sources can be utilized for novel procedures concerning fertility restoration in primarily infertile women, as well as in cancer treatment-induced fertility.

## Figures and Tables

**Table 1:** List of pluripotency, oocyte, germ cell, meiosis, primordial germ cell granulosa and theca cell-specific markers along with their functions. All of these markers have specific roles at various developmental stages.

Category	Name of Gene	Function
Pluripotency marker	Oct4	Regulates pluripotency and also acts as germ cell marker.
	Nanog	Maintains pluripotency.
	Sox2	Maintains pluripotency.
	Rex	Maintains pluripotent state.
	Ssea4	Regulates the pre-implantation development and pluripotency of human embryonic stem cells.
Oocyte-specific marker	Gdf9	Oocyte maturation marker.
	C-Mos	Oocyte maturation factor Mos.
	Zpc	Contributes to the structural integrity of oocyte and its coat.
	Stella	Involved in Chromosomal organization or RNA processing.
Germ cell-specific marker	Dazl	Primordial germ cell migration, germ stem cell proliferation differentiation, Suppresses pluripotency markers expression.
	Vasa	Germ cell determination and oocyte development.
	Blimp1	Master regulator of the foundation of the germ cell lineage and primordial germ cell formation.
	Fragilis	Thought to be involved in the initiation of homeobox genes repression in the early germ cell precursors, and pluripotency maintenance.
Meiosis-specific marker	Dmc1	Essential for meiotic recombination and meiosis regulation.
	Scp (1,2,3)	Synaptonemal complex protein (1,2,3) are required for normal centromere pairing, chromosomal synapsis during male and female germ cell development, important meiosis marker.
	Rec8	Meiotic recombination protein which participate in meiotic process (meiotic cohesion complex, sister chromatid cohesion and homologous chromosome recombination).
	Stra8	Regulation of meiotic initiation in both spermatogenesis and oogenesis.
Primordial germ cell marker	Prdm1/Blimp-1	Repress beta-interferon gene expression, plays critical role in primordial germ cells and germ cell formation.
	Prdm14	Maintain pluripotency and suppress the expression of differentiation marker genes, helps in primordial germ cell formation.
	Dppa3	Developmental pluripotency-associated protein 3 participate in epigenetic chromatin reprogramming, prevent 5mC to conversion (DNA methylation protection), the condensation of chromatin during oocytogenesis.
	Dnd1	Survival of primordial germ cells, germ cell tumor suppression in mice, apoptosis suppression.
	Nanos3	Maintain undifferentiated germ cell state, participate in human germ cell development, apoptosis suppression.
	Tcfap2c/Ap2-γ	Placenta development, primordial germ cell maintenance.
Granulosa cell marker	Foxl2	Important for ovarian development and function, granulosa cell differentiation marker, support pre-ovulatory follicles growth.
	Amh	TGF-β family member, help in the proliferation of mitotically active germ cell, folliculogenesis regulation, molecule biomarker for ovarian reserve and menopause timing.
	FshR	Expressed by granulosa cell, necessary follicular development, controls the growth and maturation of spermatogenesis and follicle formation.
	CYP19A1	Helps in estrogen biosynthesis.
Theca cell marker	LHR	Helps in follicular maturation, ovulation and luteal function.

**Table 2:** Detailed description of different species-derived, oocyte-like cells (OLCs) and primordial germ cells (PGCs) when using co-culture and follicular fluid.

Stem Cell Source		Co-Culture	FF Concentration	Final Product	Reference
Human	Ovarian stem cell	MEF layer	0	OLCs	[57]
	Wharton’s jelly	Placental cells	0	OLCs	[12]
	Theca cell	GCs	5% human	OLCs	[5]
	Menstrual blood-derived endometrial MSC	GCs	0	OLCs	[15]
	Induced Pluripotent Stem Cells	Fetal gonadal stromal cells	0	PGCs	[82]
	Embryonic stem cell	Fetal gonadal stromal cells	0	PGCs	[82]
	Embryonic stem cell	MEF layer	0	PGCs	[73]
Porcine	Female germline stem cell	GCs	5% porcine	OLCs	[83]
	Female germline stem cell	MEF layer	5% porcine	OLCs	[83]
Mouse	Skin-derived stem cell	MEF layer	0	PGCs	[21]
	Skin-derived stem cell	MEF layer	0	PGCs	[37]
	Skin-derived stem cell	Newborn mouse ovarian cells	0	OLCs	[18]
	Embryonic stem cell	Ovarian somatic cells	0	OLCs	[20]
	Embryonic stem cell	GCs	0	OLCs	[20]
	Embryonic stem cell	GCs	0	Germ-like cells	[84]
	Embryonic stem cell	New born mouse granulosa cells	0	OLCs	[17]
	Embryonic stem cell	OP9 stromal cells	0	PGC-like cells	[85]
	Ovarian stem cell	GCs	0	OLCs	[22]
	Female germline stem cell	GCs	0	OLCs	[86]
	Embryonic stem cell	MEF layer	0	PGCs	[41]

MEF: mouse embryonic fibroblast; GCs: granulosa cells; OLCs: oocyte-like cells; and PGCs: primordial germ cells.

**Table 3:** List of transfection-based female germ-specific studies, along with their description including cell source used and final research outputs.

Cell Source		Transfection	Final Product	Implication	Knockdown	Reference
Human	ASCs	BMP15	OLCs	BMP15 activation, expressed oocyte-specific markers (ZP1, ZP2, ZP3, c-kit), detection of VASA protein during later stage of induction.	x	[94]
	ESCs	NANOS3	Germ cells	Over expressed protein levels PRKSCH. No expression of OLFM2, delayed in vitro differentiation.	x	[95]
	ESCs	DAZL	Germ cells	Up-regulated DAZL, IFITM3, BMP7, HIP1R, ISYNA1, JAG2, PIDD1, RPRM. Down-regulated CXCL5, GABRP, FAM110C, LCP1.	x	[95]
	ESCs	DAZL, BOULE	Follicle-like cells	Increased DAZL and BOULE expression. down-regulation of pluripotency markers (OCT4, NANOG, PRDM14), up-regulated luciferase activity. Promoted entry into meiosis.	shRNA (DAZL)	[16]
	ASCs	BMP15	OLCs, GC	Differentiation into OLC, GC, with increased size, BMP15 activation and parthenogenetic embryo-like structures formation.	x	[11]
	UCMSCs	FIGLα	OLCs	Over expressed Figlα, stimulated germ cell-specific markers (DAZL, GDF9, ZP2)	x	[7]
	ESCs	PRDM1	PGCs	Suppressed Sox2 expression, promoted germline differentiation.	shRNA (PRDM1)	[74]
Mouse	ESCs	FIGLα	Female germ-like cells	Differentiation into germ-like cells, high expression of germ cell markers (VASA), meiotic-specific genes (STRA8, SCP3), and oocyte markers (GDF9, ZP3, FIGLα).	x	[96]
	ESCs	DAZL	Germ cells	Overexpression of DAZL. Suppression of Nanog. Induced germ cell nuclear antigen expression.	siRNA (DAZL)	[42]
	ESCs	PRDM1	Germ cells	Suppressed SOX2 expression and neural fate, increased expression of markers like PRDM1, OCT4, NANOS3, VASA and SCP3, enhances BMP4 & WNT3A induced germline differentiation.	x	[73]
	Embryonal carcinoma cells	BLIMP1, PRDM14, AP2γ	PGCs	Directed EpiLCs formation, up-regulated PGC-specification/development genes BLIMP1, PRDM14, TFAP2C, NANOS3, STELLA, down-regulated epigenetic modifier (DNMT3a, DNMT3b).	x	[71]
Porcine	Ovarian cortex	OCT4	OLCs	Oct4 overexpression. Enhanced AP activity and cell proliferation. Higher oogenesis potential.	x	[29]
	Skin-derived stem cells	DAZL	PGCs Or OLCs	Induced differentiation. Increased expression of germ cell markers (Oct4, Stella, Vasa) and meiosis markers (Scp3, Dmc1, Rec8, Stra8).	siRNA (DAZL)	[28]
Goat	Ear Pinnae cells	OCT4, SOX2, NANOG	iPSCs OLCs	Cell reprogramming, increased potency.	x	[32]

ASCs: amniotic fluid stem cells; ESCs: embryonic stem cells; UCMSCs: umbilical cord mesenchymal stem cells; OLCs: oocyte-like cells; PGCs: primordial germ cells; PRKSCH: protein kinase C substrate 80K-H; OLFM2: Olfactomedin2; IFITM3: interferon-induced transmembrane protein; BMP7: bone morphogenetic protein 7; HIP1R: huntingtin interacting protein 1 related; ISYNA1: inositol-3-phosphate synthase 1; JAG2: jagged 2; PIDD1: p53-induced death domain protein 1; RPRM: reprimo; CXCL5: chemokine ligand 5; GABRP: gamma-aminobutyric acid a receptor, Pi; FAM110C: family with sequence similarity 110, Member C; and LCP1: lymphocyte cytosolic protein 1.

**Table 4:** In vivo efficacy of mesenchymal stem cells regarding the treatment of female reproductive disorders.

Stem Cell Source		Target Disease Model	Implication	Reference
Bone marrow mesenchymal stem cell	Mouse	POF	Formed primordial follicles, increased E2 level, decreased FSH level, attained pregnant after natural breeding.	[48]
	Rabbit	POF	Increased E2 and VEGF levels, decrease FSH level, increased follicle number.	[109]
	Human	POF	After transplantation menstruation improved, one case delivered healthy full baby, increased ovarian reserve score.	[115]
Umbilical cord mesenchymal stem cell	Human	POF	Decreased serum FSH level, recovery in serum E2 and AMH levels, increase in secondary follicles, reduced ovarian cell’s apoptosis.	[112]
	Human	POI	Increased body weight, estrous cycle recovery, increase in ovarian follicles, increase in serum E2 level, decrease in serum FSH level, induced angiogenesis and cytokine expression in the ovary.	[56]
	Human	POI	Increase in ovarian weight, increased weight in E2-dependent organs, increased follicular number, decrease in serum FSH level, increase in AMH level, increased AMH expression, elevation in pregnancy rate.	[54]
Menstrual blood stem cell	Human	POF	Increased expression of ovarian markers [AMH, inhibin α/β, and follicle-stimulating hormone receptor (FSHR)], increased Ki67 expression, increased ovarian weight, increased plasma E2 level and increased number of normal follicles.	[9]
	Human	POI	Increased ovarian weight and total follicle number, estrus cycle restoration, decreased serum FSH level, increased serum E2, AMH level, reduced apoptosis, increase in the expression of AMH, DDX4 and VEGFA.	[51]
Amniotic MSC	Human	Natural ovarian aging	Increase in all stage follicle number, recovery in E2 and AMH levels, decreased FSH levels, promoted the proliferation rate, high expression of ovarian and granular cell markers (AMH, FSHR, FOXL2, CYP19A1).	[111]
	Human	POF	Recovered estrus cycle, increase in estrogen level, decrease in FSH levels, increase in ovarian index, fertility rate and population of follicles at different stages.	[53]
Chorionic MSC	Human	POF	Decreased serum FSH level, increased serum E2 level and number of follicles, restored estrous cycle, increased oocyte population.	[50]
Adipose-derived MSC	Human	Damaged ovarian	Induced angiogenesis, increase in ovarian follicles, corpus luteal and also in number of litters.	[113]
Endometrial MSC	Human	POF	Increased body weight, restored estrus cycle, re- established fertility, MSC infiltration to damaged ovarian tissue and differentiated into granulosa cell, improved renewal of germline stem cells.	[110]
Skin-derived MSC	Mouse	POF	Increased body weight, increased weight of reproductive organs, restored fertility, reduced pro-inflammatory cytokines (TNF-a, TGF-b, IL-8, IL-6, IL-1b, and IFN γ), increased expression of genes Nobox, Nanos3, and Lhx8 genes in the transplanted ovaries.	[46]

POF: premature ovarian failure; POI: premature ovarian insufficiency; FSH: follicular stimulating hormone; LH: luteinizing hormone; AMH: anti Mullerian hormone; and E2: estradiol.

**Table 5:** Description about the list of ongoing clinical trials that are associated with the use of stem cells in curing female reproductive disorders. All of these details were taken from https://stemcellsportal.com/clinical_trials_infertility.

Pathological Condition	Cell Therapy	Estimated Enrollment	Intervention/ Treatment	Cliniclatrials.Gov Identifier	Outcome Measures
Women infertility with thin endometrium or endometrial scarring.	UCMSCs loaded in collagen scaffold	50 participants	UCMSC-based therapy	NCT03592849	Evaluation of endometrial thickness, pregnancy rate, living intrauterine fetus, live birth rate, endometrial blood flow, menstrual blood volume and adversities such as infection, allergies, abdominal pain post-operation.
Women with POI	AECs	20 participants	Minimally invasive implantation AECs used with ultrasound guidance	NCT03207412	Evaluation of serum FSH, primordial egg follicles number, menstrual cycle and menstrual period, LH, AMH and E2, ovarian volume.
Repeated IVF failure women with atrophic endometrium	Autologous BMSCs	46 participants	Transplantation of BMSCs	NCT03166189	Evaluation of endometrial receptivity, endometrial thickness, pregnancy rate, adversities such as side effects, abdominal discomfort and patient’s tolerance.
Women with POF	Autologous BMSCs	33 participants	Transplantation of BMSCs	NCT02696889	Evaluation of FSH, AMH, E2 level, menstruation resumption, pregnancy achievement.
Women with POF	Autologous AMSCs	9 participants	Intra-ovarian injection of AMSCs	NCT02603744	Evaluation of FSH, AMH, antral follicle number and volume, menstruation recurrence rate and pregnancy rate.
Women infertility with intrauterine adhesions	UCMSCs loaded in collagen scaffold	26 participants	UCMSC-based therapy	NCT02313415	Evaluation of live birth rate, intrauterine adhesion reduction, change of endometrial thickness, menstrual blood volume.
Women infertility with intrauterine adhesions and endometrial dysplasia	Autologous BMSCs loaded in collagen scaffold	30 participants	BMSC-based therapy	NCT02204358	Evaluation of intrauterine scar reduction, endometrial thickness, menstrual blood volume and pregnancy rate.
Women with POF	Autologous BMSCs	60 participants	Transplantation of BMSCs	NCT02062931	Evaluation of FSH, AMH, E2 level, hormonal, clinical, ultra sound, menopausal symptoms and pregnancy rate.

UCMSCs: umbilical cord mesenchymal stem cells; AECs: amniotic epithelial cells; BMSCs: bone marrow-derived mesenchymal stem cells; AMSCs: adipose mesenchymal stem cells; POF: pre-mature ovarian failure; POI: pre-mature ovarian insufficiency; FSH: follicular stimulating hormone; LH: luteinizing hormone; AMH: anti Mullerian hormone; and E2: estradiol.
