# Erratum: Bharti D., et al. In Vitro Generation of Oocyte Like Cells and Their In Vivo Efficacy: How Far We have been Succeeded. *Cells* 2020, *9*, 557

**DOI:** 10.3390/cells9051262

**Published:** 2020-05-20

**Authors:** Dinesh Bharti, Si-Jung Jang, Sang-Yun Lee, Sung-Lim Lee, Gyu-Jin Rho

**Affiliations:** 1 Department of Theriogenology and Biotechnology, College of Veterinary Medicine, Gyeongsang National University, Jinju 52828, Korea; bhartidinesh54@gnu.ac.kr (D.B.); sjjang@gnu.ac.kr (S.-J.J.); sy_lee@gnu.ac.kr (S.-Y.L.); sllee@gnu.ac.kr (S.-L.L.); 2 Research Institute of Life Sciences, Gyeongsang National University, Jinju 52828, Korea

The wrong grant number was erroneously entered in the original manuscript and needs to be changed from NRF-2017R1D1A1B03035677 to NRF-2019R1I1A3A01060073 in [1]. The authors therefore wish to replace the funding section with the following:

**Funding:** This work was supported by a grant from the National Research Foundation (NRF-2019R1I1A3A01060073) and from Stem Centric Co. Ltd. in the Republic of Korea.

The authors would like to apologize for any inconvenience caused to the readers by this change. The changes do not affect the scientific results. The manuscript will be updated and the original will remain online on the article webpage, with a reference to this Erratum.
